# Predicting the Origins of Anti-Blood Group Antibody Specificity: A Case Study of the ABO A- and B-Antigens

**DOI:** 10.3389/fimmu.2014.00397

**Published:** 2014-08-22

**Authors:** Spandana Makeneni, Ye Ji, David C. Watson, N. Martin Young, Robert J. Woods

**Affiliations:** ^1^Complex Carbohydrate Research Center, University of Georgia, Athens, GA, USA; ^2^Human Health Therapeutics, National Research Council Canada, Ottawa, ON, Canada; ^3^School of Chemistry, National University of Ireland, Galway, Ireland

**Keywords:** molecular docking, MD simulations, blood group antigens, antibody specificity, GLYCAM, AMBER

## Abstract

The ABO blood group system is the most important blood type system in human transfusion medicine. Here, we explore the specificity of antibody recognition toward ABO blood group antigens using computational modeling and biolayer interferometry. Automated docking and molecular dynamics simulations were used to explore the origin of the specificity of an anti-blood group A antibody variable fragment (Fv AC1001). The analysis predicts a number of Fv-antigen interactions that contribute to affinity, including a hydrogen bond between a His^L49^ and the carbonyl moiety of the GalNAc in antigen A. This interaction was consistent with the dependence of affinity on pH, as measured experimentally; at lower pH there is an increase in binding affinity. Binding energy calculations provide unique insight into the origin of interaction energies at a per-residue level in both the scFv and the trisaccharide antigen. The calculations indicate that while the antibody can accommodate both blood group A and B antigens in its combining site, the A antigen is preferred by 4 kcal/mol, consistent with the lack of binding observed for the B antigen.

## Introduction

Since its discovery in 1900 (1), the ABO blood group system has played a crucial role in defining human blood and tissue compatibility. The blood type of an individual indicates the presence or absence of relevant antigens and antibodies. The three blood types share a core oligosaccharide antigen (H), and based on the glycosyl transferases inherited, different antigens are synthesized (2–4); type A transferase adds a terminal non-reducing *N*-acetylgalactosamine (GalNAc) residue; type B transferase adds galactose (Gal), whereas individuals with blood group O retain the unmodified H antigen. During the first years of life, the immune system forms antibodies upon exposure to non-self antigens from various exogenous factors. Thus an A-type individual will have circulating antibodies specific for the B-antigen, and vice-versa. The high degree of specificity is notable given that the only difference between the structures of the A- and B-antigens is the replacement of an acetamido moiety (in A) with a hydroxyl group (in B). Because of the presence of circulating antibodies, a mismatched blood transfusion or organ transplant can lead to hyperacute immune response and death (5, 6). Additionally, under certain circumstances, incompatibilities in blood groups between mother and child can trigger the mother’s immune system to produce antibodies against the fetus, causing hemolytic disease (7).

Alterations in the structures of the ABO antigens often occur during carcinogenesis and therefore they have also been considered tumor markers (8, 9). Recently, strong correlations have been established between the presence of particular ABO and Lewis antigens and susceptibility to infectious diseases, such as *Helicobacter pylori*, norovirus, and cholera (10), wherein the blood group antigens can be exploited as receptors for bacterial and viral adhesion. Conversely, it has been suggested that endogenous anti-blood group antibodies can recognize blood-group-like carbohydrate antigens on pathogen surfaces, conferring protection against infection (11).

Despite their clinical importance, relatively little is known about the structural basis for these highly specific antibodies–antigen interactions. Although X-ray crystallography has been used to characterize antibody–carbohydrate complexes, the generally enhanced flexibility and conformational heterogeneity of oligosaccharides detracts from the ability to generate co-crystals (12). Additionally, anti-carbohydrate antibodies bind to their antigens with an affinity that is 3–5 orders of magnitude lower than typical antibodies that bind to protein or peptide antigens. Difficulties in generating 3D structures for carbohydrate–antibody complexes have led to the increasing use of theoretical structure prediction methods (13, 14), which, while convenient, are prone to predicting false positives due to inaccuracies in pose scoring functions (15) and to the omission of carbohydrate conformational preferences (16).

In this study, we examined the structural origin of the antigenicity (the specificity and affinity) of a monoclonal antibody raised against blood group A (BGA) antigen, for which an apo structure of the single-chain variable fragment (scFv AC1001) has been reported (17). The specificity data from screening two independent glycan arrays [Consortium for Functional Glycomics (v4.0, request ID: 1808) and from the group of Jeff Gildersleeve] confirmed that the scFv displayed no detectable binding to any B-antigens and only bound to BGA-containing glycans. To provide a structural interpretation for the specificity of AC1001 for BGA over blood groups H (BGH) and B (BGB), we generated a 3D model of the immune complex using molecular docking and refined it by molecular dynamics (MD) simulation. Despite its limitations, molecular docking, with or without additional experimental constraints, such as from NMR data, is often the only approach that may be employed to generate the structure of a ligand–protein complex, in the absence of direct crystallographic data. To enhance the success rate, a recent carbohydrate conformational energy function (16) was employed with AutoDock VINA (18), which quantifies the conformational preferences of oligosaccharides based on their glycosidic torsion angles. MD simulations (50 ns) were subsequently performed to ensure that the docked complexes were stable under physically realistic conditions, and in that event, the MD data were employed in binding free energy calculations. A particular advantage of MD-based energy calculations is that they provide statistically converged values that may be partitioned into contributions from individual residues in the protein and ligand (19).

## Materials and Methods

### Cloning, expression, and purification of scFv

An scFv gene containing a short linker (RADAA) and the Leu 103H Val mutation (17), with a His_6_ tag, was assembled by PCR and cloned into the phagemid pSK4. The construct was maintained in *Escherichia coli* TG1 cells. Cells from positive clones, as judged by DNA sequence analysis, were grown in minimal media, induced, and subjected to periplasmic extraction. The scFv dimer was purified from the extract by Ni^2+^ immobilized metal affinity chromatography, by elution with an imidazole gradient.

### Biolayer interferometry

Affinity measurements were performed on a biolayer interferometer (Octet Red96, ForteBio). Data were processed using the Data Acquisition and Analysis 8.0 software (ForteBio), and kinetic binding constants were determined from a 1:1 binding model using the OriginPro software (OriginLab). The scFv was immobilized on an amine reactive second-generation (AR2G) biosensor (Lot No. 1311212, ForteBio). The BGA trisaccharide was analyzed as the conjugate to bovine serum albumin (BSA–BGA) and was dissolved in an analysis buffer containing 10 mM HEPES, 150 mM NaCl, 3.4 mM EDTA, and 0.005% Tween 20 at a range of pH values (5, 5.5, 6, 6.5, and 7). A BSA–Le^X^ trisaccharide conjugate (Prod. No. NGP0302, V-Labs, Inc.) and BSA (Prod. No. 23209, Pierce Thermo Scientific, Rockford, IL, USA) were used as negative controls. Details of the biolayer interferometry (BLI) conditions are provided in Supplemental Material.

### Automated docking

Docking was performed using AutoDock VINA (18) with 20 docked poses generated for each experiment. The protein and the ligand files were prepared using Autodock tools (ADT) (20) with Gassteiger (21) partial atomic charges assigned to both the protein and ligand residues. The crystal structure of the scFv (PDB ID: 1JV5) was employed, together with a 3D structure of BGA obtained from the GLYCAM-Web server (www.glycam.org). Crystal waters were removed prior to docking and hydrogen atoms were added to the protein using ADT, whereas hydrogen atoms in the ligand were assigned from the GLYCAM residue templates. The glycosidic ϕ and φ torsion angles were allowed to be flexible during docking, as were all the hydroxyl groups. The protein was maintained rigid. The docking grid box (dimensions: 26.25 Å × 26.25 Å × 37.5 Å) was centered relative to the complementarity determining regions (CDRs) of the antibody as described previously (16). For the mutational-docking approach, Trp^H100^ was mutated to Ala by deleting the side-chain atoms of the Trp residue in the crystal structure, followed by processing with the tleap module in AMBER (22). Ala^H100^ was reverted back to Trp by restoring the crystal coordinates of the side chain of Trp^H100^. The docked poses from the mutational approach were filtered based on the clashes with the reverted Trp. Poses in which the clashes could not be eliminated by implicit energy minimization (details are in the “MD simulations” section) were rejected. Ligand conformations of all the docked poses from both the flexible and mutational-docking approaches were scored using the recently reported carbohydrate intrinsic (CHI) energy scoring function (16). Any conformations with total CHI-energies >5 kcal/mol were rejected. The BGB complex was generated directly from that generated for BGA by simple replacement of the NAc group by an OH group.

### MD simulations

All the MD simulations were performed with the GPU implementation of the pmed code, pmed.cud_SPDP (23), from AMBER12 (22). The calculations employed the ff99SSB (24) parameters for the protein and the GLYCAM06h (25) parameters for the carbohydrate. For the BGA, BGB–scFv complex simulations, an implicit solvent energy minimization (5000 steps of steepest descent followed by 5000 steps of conjugate gradient), were performed to optimize the side-chain positions of the reverted Trp residue. During this minimization, the backbone atoms of the framework regions were restrained with a 5 kcal/mol Å^2^ while the CDRs and the ligand were allowed to be flexible. The systems were then solvated in a cubic water box [120 Å per side, with a TIP3P water (26)]. Each system was energy minimized using explicit solvent (10,000 steps of steepest descent, 10,000 steps of conjugate gradient). During this energy minimization, the protein residues were restrained with a force constant of 100 kcal/mol Å^2^ allowing only the solvent and ligand to relax. This minimization was followed by heating from 5 to 300 K over the course of 50 ps at constant volume. Production MD simulations were performed for 50 ns at constant pressure (NPT ensemble) with the temperature held constant at 300 K using a Langevin thermostat. During the heating and the production MD, the backbone atoms of the protein were restrained with a force constant of 5 kcal/mol Å^2^, with the protein side chains and ligand atoms allowed to be flexible. The backbone atoms were restrained in order to ensure that the protein fold remained stable during the course of the simulation. For the BGA trisaccharide MD simulation, the system was solvated in a cubic water box (120 Å per side, with a TIP3P water) and energy minimized using explicit solvent (5000 steps of steepest descent, 5000 steps of conjugate gradient). This was followed by heating from 5 to 300 K for a period of 50 ps at constant volume. Production MD simulations were performed for 50 ns at constant pressure (NPT). During the minimization, heating, and production MD simulations, there were no restraints placed on the trisaccharide. For both BGA, BGB–scFv complexes and BGA trisaccharide simulations, all covalent bonds involving hydrogen atoms were constrained using the SHAKE (27) algorithm, allowing a time step of 2 fs. A non-bonded cut-off of 8 Å was used and long-range electrostatics were employed using the particle mesh Ewald (PME) method (28). Snapshots were collected at 1 ps intervals for subsequent analysis.

### Analysis

The stability of the complexes was assessed by monitoring the root-mean-squared-displacement (RMSD) of the ligand position, the glycosidic torsion angles, the ring conformations, and the protein–ligand hydrogen bonds. All these values except for the ring conformation analysis were generated using the ptraj module of AMBERTOOLS 12 (29). Ligand RMSD values were calculated for the ring atoms, relative to the first time step of the simulation. Hydrogen bond interactions between the protein and the ligand were measured with distance and angle cut-off values of 3.5 Å and 120°, respectively. The ring conformations of each individual residue in the ligand during the course of simulation were analyzed using the recently reported BFMP method (Makeneni et al., submitted). Binding free energies were calculated with the MM-GBSA (30, 31) module in AMBERTOOLS12. All the water molecules were removed prior to the MM-GBSA calculation, and desolvation free energies were approximated using the generalized born implicit solvation model (igb = 2) (32).

## Results and Discussion

### Docking analysis

**Figure 1:**
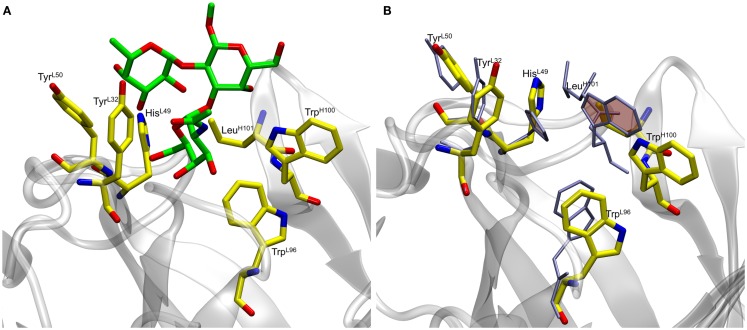
**(A)** Docked antigen A (green) from preliminary docking experiments with residues lining the binding pocket (shown in yellow). The antibody is shown in gray. **(B)** Residues lining the binding pocket before (yellow) and after (ice blue) the 50 ns MD simulation. Residues His^L49^ and Trp^H100^ (shaded rings) form stacking interactions during the course of the simulation thereby causing the ligand to become unstable.

In preliminary experiments, docking to the rigid scFv structure yielded complexes that failed to remain stable during subsequent 10 ns MD simulations (Table S1 in Supplementary Material). The spontaneous dissociation of the complex during MD simulation suggested that the docking had failed to detect the correct, high affinity, pose (33). Upon inspection of the MD data, it was observed that light chain residue His49 (His^L49^) forms a stacking interaction with heavy chain residue Trp100 (Trp^H100^), which occupies a large volume of the presumed binding site, potentially preventing deeper penetration of the ligand (Figure 1).

**Figure 2:**
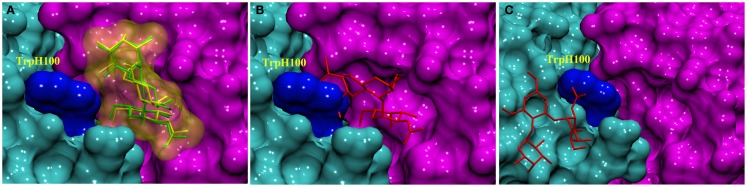
**Docked complexes of BGA (stick structure) in the scFv binding site (heavy and light chains shown as solvent accessible surfaces in cyan and pink, respectively, the Trp^H100^ surface is shown in dark blue)**. **(A)** The stick structures in green and yellow represent the best-docked poses from the Trp^H100^-mutagenesis and the flexible residue docking approaches, respectively. **(B)** An example of a docked pose (red) that was eliminated on the basis of clashes ensuing from the Ala^H100^Trp mutation. **(C)** An example of a docked pose (red) that was eliminated on the basis of the orientation of the ligand in the binding pocket.

As Trp residues can also form stacking interactions with the apolar face of monosaccharides in antibody complexes (34), we hypothesized that the trisaccharide ligand might compete for formation of such an interaction with Trp^H100^. For example, the galactose (Gal) residue in a *Salmonella* trisaccharide antigen stacks against Trp^L93^ in the complex with Fab Se155-4 (34). In addition, in the same complex, Trp^H33^ stacks against the C-6 position in the 6-deoxy sugar Abequose. The BGA antigen contains GalNAc and a 6-deoxy monosaccharide (fucose, Fuc), thus a revised docking experiment was sought that would permit the formation of such interactions with the aromatic residues in the binding pocket. Thus, two alternative docking experiments were designed: in the first, the side-chain torsion angles of Trp^H100^ were allowed to be flexible during docking (termed flexible residue docking); while in the second, Trp^H100^ was mutated to Ala prior to docking, and then reverted back to Trp after docking (mutational residue docking). The docked poses were filtered based on three criteria. First, poses in which the GalNAc was not located within the binding pocket were eliminated (Figure 2C). This criterion was adopted based on the results from two array screenings, which indicated that the antibody interacts exclusively with the BGA antigens (Tables S2 and S3 in Supplementary Material) and because the only structural difference between BGA and BGB is the presence of the NAc moiety in the former. Therefore it was hypothesized that the ability of the antibody to discriminate between these two antigens would be dependent on interactions with this residue. Second, in the case of the mutational approach, poses were rejected if the Ala-Trp mutation led to irreconcilable steric clashes with the antigen (Figure 2B). All the docked poses obtained from each of these approaches were then scored using a CHI scoring function. After applying these criteria, both docking approaches identified essentially equivalent antigen poses (0.48 Å RMSD between ligand positions) (Figure 2A), in which the C-6 atom of the GalNAc forms a CH/π stacking interaction with the Trp^H96^. This complex was selected for further analysis by MD simulation.

### Structural stability of the immune complexes

#### Blood group A

The final docked model of the blood group antigen A bound to the antibody remained stable during the course of a 50 ns simulation based on the RMSD of the ring atoms of the ligand, which remained between 2 and 4 Å over the course of the simulation (Figure 5). An analysis of the ring conformational preferences showed that all three residues in the trisaccharide remained in the ^4^C_1_ chair conformations. The ϕ- and ψ-glycosidic torsion angles for the GalNAcα(1,3)Gal (ϕ_1_, ψ_1_) and Fucα(1,2)Gal (ϕ_2_, ψ_2_) linkages were monitored throughout both the simulations (BGA–scFv complex and BGA trisaccharide in solution) and the average values were found to be in agreement with the values observed for the same trisaccharide in the complex with *Dolichos biflorus* lectin as well as the conformations of the trisaccharide in solution (35) (Table 1). The stacking interactions between the GalNAc and Trp^H96^ interactions were characterized by the angle (θ) between the normals to the ring planes, and the distance (*R*) between their centroids (36). For an ideal stacking conformation, θ should be around 180° or 0°, and for CH/π, it should be around 90°. The average θ value was close to the latter at 108° (with a standard deviation of 9°) at a distance of 6.5 Å.

**Table 1:** **Comparison of glycosidic torsion angles between experimentally observed values and average values obtained from the MD simulations**.

	(ϕ_1_, φ_1_)^a^		(ϕ_2_, φ_2_)^b^	
	Experimental	Theoretical	Experimental	Theoretical
BGA trisaccharide	62° < ϕ_1_ < 82°, 61° < φ_1_ < 74°	−68 ± 14°, 51 ±25°	−77° < ϕ_2_ < −67°, −109° < φ_2_ < −86°	−69 ± 11°, −101 ± 26°
BGA–scFv complex	68°, 77°	82 ± 11°, 68 ± 7°	−68°, −90°	−69 ± 8°, −113 ± 10°

*^a^Glycosidic torsion angles for the GalNAc**α**(1,3)Gal (ϕ_1_, φ_1_)*.

*^b^Torsion angles for Fuc**α**(1,2)Gal (ϕ_2_, φ_2_)*.

**Figure 3:**
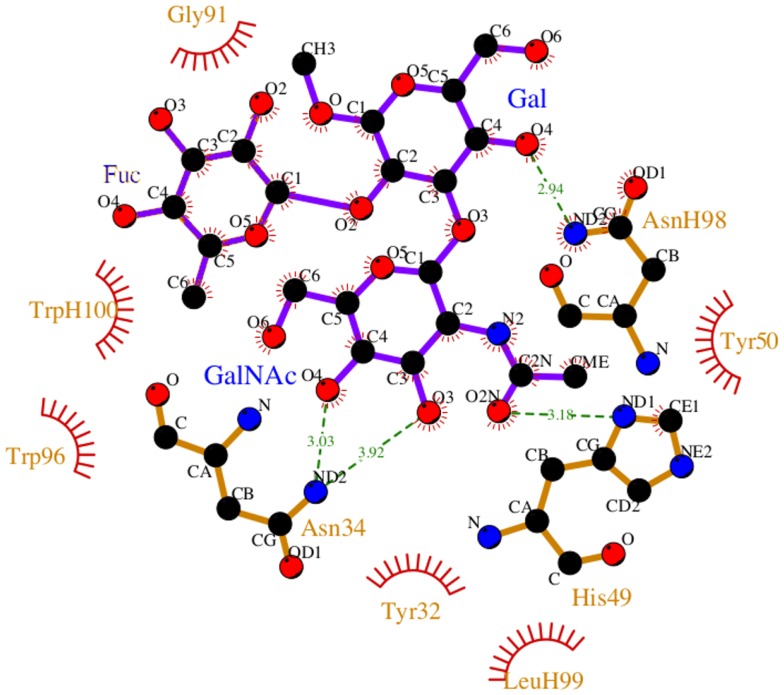
**Non-bonded interactions between the BGA and Fab AC1001 [prepared using LigPlot (38)]**. The structure represents a single frame of the MD simulation that is closest to the average RMSD of the structure during the simulation. Unless shown with an H, all residues are from VL.

**Figure 4:**
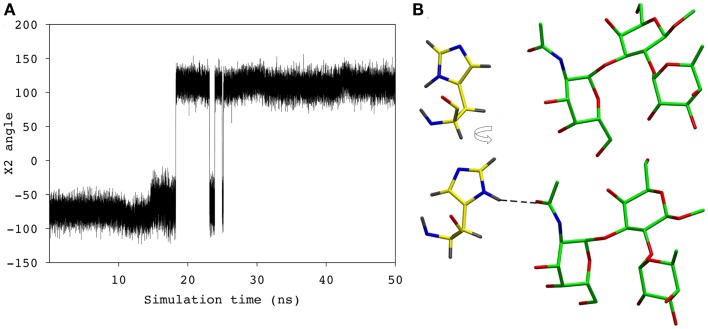
**(A)** The χ_2_ angle of the His^L49^ during the course of the simulation. **(B)** His^L49^ (shown in yellow) during the first 18 ns of the simulation (top) and the remainder of the simulation (bottom).

During the course of the MD simulation, the side chain of His^L49^ was observed to flip from its initial orientation (χ_2_ = 〈-73〉) to (〈115〉) in which it could form a hydrogen bond with the N-acetyl group of the GalNAc residue (Figures 3 and 4; Table 2). This interaction remained stable for the remainder of the 50 ns simulation. This side-chain flip may represent an example of induced fit during ligand binding, however, at the resolution of the present X-ray data (2.2 Å), it is not possible to reliably discriminate between histidine χ_2_ rotamers (37).

**Table 2:** **Hydrogen bonds between BGA and the scFv during the MD simulation**.

Donor		Acceptor		MD period: 0–18 ns		MD period: 18–50 ns	
				Distance^a^ (Å)	Occupancy (%)	Distance (Å)	Occupancy (%)
GalNAc	O3	Asn^L34^	Hδ1	3.1 (0.18)^c^	67	> 3.5	–
	O4	Asn^L34^	Hδ1	3.1(0.22)	32	3.0 (0.17)	77
	O2N	His^L49^	Hδ	>3.5	–	2.9 (0.16)	91
Gal	O4	GalNAc	H2N	3.2 (0.17)	65	3.2 (0.17)	31
	O4	Asn^H98^	Hδ1	3.1 (0.18)	45	3.1 (0.17)	41

*^a^Standard deviations in parentheses*.

#### Blood group B

**Figure 5:**
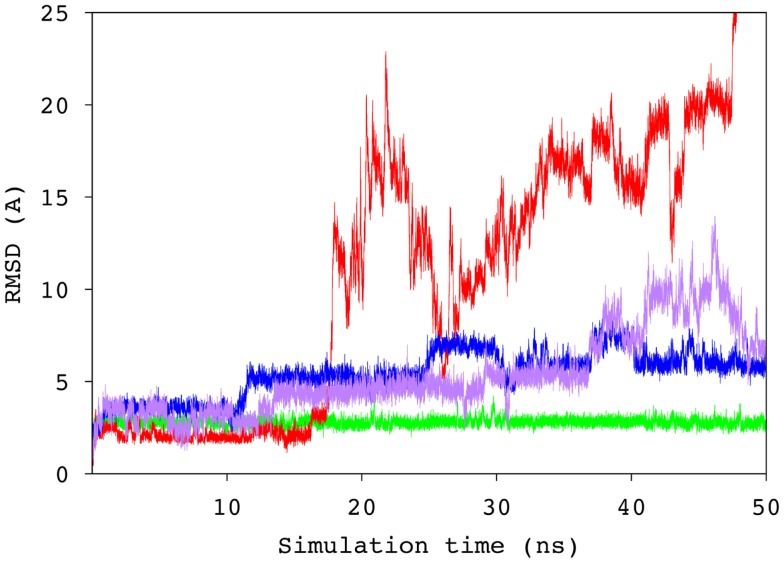
**Time series of the RMSD values for the ring atoms of the BGA (green) and BGB (from three independent simulations, blue, purple, and red) antigens, relative to the starting conformation of the complex**.

To probe the specificity of the antibody for antigen B, the scFv was screened experimentally against an array of neoglycoconjugates including ABO and related blood group antigens. The screening confirmed the exclusive specificity of the antibody for BGA-related antigens (Tables S2 and S3 in Supplementary Material). Computational carbohydrate grafting (39) of the relevant glycans from the array onto the bound BGA trisaccharide in the scFv complex confirmed that all of the BGA- and BGB-related glycans could be accommodated in the binding pocket (Table S3 in Supplementary Material). Therefore, the lack of binding of the BGB-glycans does not appear to be due to steric collisions, but rather to the loss of affinity arising from the absence of the NAc group in the BGA congeners. MD simulation of the BGB–scFv complex was employed to examine the effect of the loss of the NAc moiety on the stability and affinity of the structural difference in the antigens on the stability and affinity of the putative immune complex. Despite the fact that the MD simulations of the two complexes (BGA and BGB) were started with the antigens aligned in identical binding modes, the BGB antigen dissociated from the antibody after a relatively short simulation period of 10 ns. In order to eliminate the possibility that this instability arose due to artifacts from the conversion of the BGA to BGB antigen, two additional simulations were performed with independent initial atomic velocities. In both cases, the ligand appeared to dissociate from the antibody after approximately 10 ns (Figure 5). To enable comparison with the BGA complex, only the data from the initial stable 10 ns period of the BGB complex were chosen for analysis.

**Figure 6:**
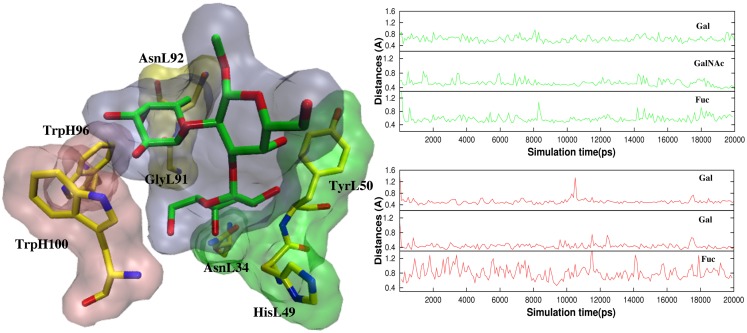
**(Left)** The antigens are flanked by Tyr^L50^, Asn^L34^, and His^L49^ (Group 1, green surface), and Trp^H100^ and Trp^L96^ (Group 2, pink surface). Fuc interacts with Gly^L91^ and Asn^L92^ (Group3, yellow surface). **(Right)** Atomic fluctuations of residues Gal, GalNAc/Gal (BGA/BGB), and Fuc as a function of time.

In antigen–scFv complexes, the Gal or GalNAc residues are flanked by residues Tyr^L50^, Asn^L34^ and His^L49^ on one side of the antigen (Group 1) and residues Trp^H100^ and Trp^L96^ (Group 2) on the other; the Fuc interacts with Gly^L91^ and Asn^L92^ (Group 3) (Figure 6). In contrast to the case of the BGA antigen, in the BGB–scFv simulation His^L49^ does not form a stabilizing interaction with the terminal Gal residue. Additionally, the Gal and Fucl residues display enhanced flexibility owing to the loss of stabilizing interactions with residues from Groups 2 and 3.

### Involvement of His^L49^ in binding affinity

**Figure 7:**
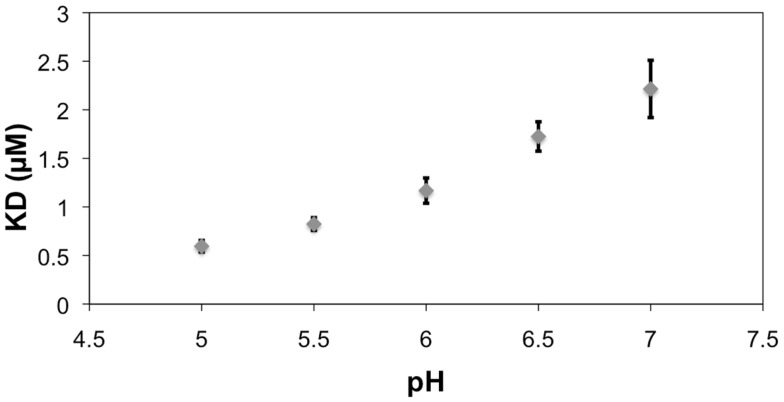
**The reference (BSA)-subtracted pH dependence of the apparent *K*_D_ for the interaction between scFv AC1001 and the BSA–BGA conjugate**. Error bars are derived from replicates of five measurements. Note, the p*K*a of histidine is 6.04 (40).

All histidines in the scFv were protonated by default for modeling with a hydrogen atom at the δ nitrogen position. During the MD simulation of the BGA–scFv complex, the χ2 angle of His^L49^ flips (-73° to 115°) enabling a hydrogen bond to form with the carbonyl moiety of the NAc group in the GalNAc residue in BGA, which would be expected to be significant for enhancing the stability of the BGA–scFv complex. In the BGB complex, the same His^L49^ forms an interaction with the non-terminal Gal residue. The interaction with HisL^49^ suggests that there might also be a pH dependence on binding; at lower pH all histidines would be positively charged, potentially enhancing the strength of the His^L49^–BGA hydrogen bond, leading to higher binding affinity. This prediction was confirmed by BLI measurements, which showed a marked decrease in the apparent *K*_D_ as the pH dropped below the p*K*_a_ of histidine (Figure 7). It should be noted that this protonation would not be localized to His^L49^ nevertheless, no enhanced non-specific binding was observed at low pH for either BSA or BGA–Le^x^ (Figures S1–S3 in Supplementary Material), supporting a role for a direct interaction between His^L49^ and the BGA antigen.

### Binding energy analysis

A per-residue decomposition of the interaction energies in the immune complexes indicated that, in the case of BGA, the GalNAc residue contributed 25% (-8.1 kcal/mol) toward the binding energy, compared to a reduced (-4.3 kcal/mol) contribution from the corresponding Gal residue in BGB (Table 3). This loss of 4 kcal/mol of interaction energy is the predominant difference between the two antigens, and would be enough to reduce the affinity by nearly 800-fold, consistent with the lack of apparent binding of the BGB analogs in the glycan array screening. In addition, this analysis identified the residues that contributed significantly toward antigen binding.

**Table 3:** **Key^a^ per-residue contributions toward the energy for the BGA and BGB–scFv complexes**.

Residue	vdW	Electrostatic	Polar desolvation	Non-polar desolvation	Total
**Antibody**					
Tyr^L32^	−4.2^b^	−1.4	2.1	−0.5	−4.0
	−3.0^b^	−1.7	2.0	−0.5	−3.2
Gly^L91^	−0.8	−4.8	3.2	−0.2	−2.5
	–	–	–	–	–
Trp^H100^	−2.5	−1.2	1.7	−0.3	−2.2
	−2.8	−1.0	1.2	−0.4	−2.9
Trp^L96^	−1.4	−1.1	0.7	−0.2	−2.0
	−0.7	−0.3	0.2	−0.1	−0.9
Asn^L34^	−0.8	−1.6	0.9	−0.1	−1.6
	−0.6	−3.7	1.5	−0.1	−2.9
Asn^H98^	−2.2	−2.9	4.0	−0.5	−1.5
	−2.8	−1.6	3.8	−0.5	−1.1
Asn^L92^	−1	−2.9	2.6	−0.2	−1.5
	–	–	–	–	–
Tyr^L50^	−1.6	−0.9	1.1	−0.1	−1.5
	−1.7	−1.0	1.4	−0.1	−1.5
Leu^H99^	−1.4	−1	1	−0.1	−1.4
	−1.4	−0.3	0.8	−0.1	−1
His^L49^	−0.6	−3.3	2.5	−0.1	−1.4
	−1.3	−2.8	2.5	−0.2	−1.8
Thr^L93^	−0.5	−0.6	0.6	0.0	−0.5
	–	–	–	–	–
**Subtotal**	−17.1	−20.5	20.5	−2.2	−19.3
	−11.3	−12.4	13.4	−2	−15.3
**Antigen**
Gal	−3.1	−0.4	1.2	−0.4	−2.7
	−6.2	−3.7	5.2	−0.9	−5.6
GalNAc/Gal	−13.1	−12.5	19.6	−2.1	−8.1
	−10.1	−8.2	15.8	−1.7	−4.3
Fuc	−4.0	−9.7	12.2	−0.8	−2.3
	−2.5	−1.2	4.5	−0.4	0.4
**Ligand total**	−20.2	−22.6	32.9	−3.2	−13.2
	−18.9	−13.2	25.4	−1.4	−9.6

*^a^Key residues defined as those that contribute >0.5 kcal/mol to the total interaction energy for either the BGA or BGB in the complexes. Only the initial stable 10 ns period of the BGB simulation was employed, whereas the entire 50 ns trajectory for BGA was analyzed*.

*^b^Upper row, values for BGA; lower, BGB*.

In the BGA–scFv complex, residues from CDR L3 make the maximum contributions to binding (Gly^L91^ + Trp^L96^ + Asn^L92^ + Thr^L93^ = -7.2 kcal/mol) followed by H3 (Asn^H98^ + Trp^H100^ + Leu^H99^ = -5.5 kcal/mol), L1 (Tyr^L32^ + Asn^L34^ = -4.5 kcal/mol), and L2 (Tyr^L50^ = -1.02 kcal/mol). In contrast, in the case of BGB, the same residues from L3 contribute less than a total of 1 kcal/mol to the interaction energies. The most significant single residues are Tyr^L32^, Gly^L91^, Trp^H100^, and Trp^L96^, which each contributes more than 2 kcal/mol and together account for 50% of the total affinity. Residues Gly^L91^ and Asn^L92^ that form hydrogen bonds with the Fuc residue together contribute -4.0 kcal/mol to the binding of BGA, but fail to make any stable interactions in the BGB simulation and therefore contribute negligibly to the affinity. It is these interactions that provide the predominant contributions to the preferential binding of the BGA antigen. While in the BGB complex, His^L49^ does not form any stable hydrogen bonds with the terminal Gal, it is able to form new, albeit transient, interactions with the non-terminal Gal for 30% of the stable simulation period. Therefore, while the per-residue contribution values indicate that His^L49^ makes a contribution greater than -1.5 kcal/mol in both cases, the interactions it forms in BGA are more stable when compared to the interactions in BGB.

## Conclusion

In this study, 3D models of the BGA and BGB trisaccharides in complex with scFv AC1001 were generated that provided a detailed atomic level rationalization of the interactions and dynamics responsible for antigen specificity. Quantification of the binding affinities identified key residues in the binding site that are predicted to contribute to specific and non-specific interactions with each antigen and led to the confirmed prediction of enhanced binding at lower pH. The spontaneous dissociation of antigen B from the scFv–BGB complexes (in three different simulations) indicated that MD simulations confirm the known preference of this antibody for the A antigen, and support a role for MD simulations in overcoming limitations associated with ligand docking. The present study illustrates that integration of multiple experimental (affinity measurements, glycan array screening, and crystallography) and theoretical (ligand docking, MD simulation, and energy decomposition) methods provides a powerful platform for predicting the origin of antibody–carbohydrate specificity.

## Author Contributions

Spandana Makeneni, N. Martin Young, and Robert J. Woods conceived and designed the experiments. Spandana Makeneni, Ye Ji, and David C. Watson performed the experiments. Spandana Makeneni, Ye Ji, and Robert J. Woods analyzed the data. Spandana Makeneni, Ye Ji, N. Martin Young, David C. Watson, and Robert J. Woods contributed reagents/materials/analysis tools. Spandana Makeneni, N. Martin Young, and Robert J. Woods wrote the paper.

## Conflict of Interest Statement

The authors declare that the research was conducted in the absence of any commercial or financial relationships that could be construed as a potential conflict of interest.

## Supplementary Material

Included in the supplemental information are details and raw data from the BLI experiments. Results from screening of the scFv against both CFG and carbohydrate arrays (Dr. Jeffery Gildersleeve’s group) are also presented in the supplemental material. The Supplementary Material for this article can be found online at http://www.frontiersin.org/Journal/10.3389/fimmu.2014.00397
