# Supplementary material for: Predicting the Origins of Anti-Blood Group Antibody Specificity: A Case Study of the ABO A- and B-Antigens
Source: Front Immunol. 2014 Aug 22;5:397. doi: 10.3389/fimmu.2014.00397 (PMC4141161; doi:10.3389/fimmu.2014.00397)
Supplement: Supplementary file 1 [file Data_Sheet1.DOCX]

***Supplementary Material***

**Predicting the origins of anti-blood group antibody specificity: A Case Study of the ABO A- and B-antigens**

**Spandana Makeneni^1^, Ye Ji^1^, David C. Watson^2^, N. Martin Young^2^, Robert J. Woods^1,3^**

^1^Complex Carbohydrate Research Center, University of Georgia, 315 Riverbend Road, Athens, GA 30602, USA

^2^Institute for Biological Sciences, National Research Council, 100 Sussex Drive, Ottawa, ON K1A 0R6, Canada

^3^School of Chemistry, National University of Ireland, Galway, University Road, Galway, Ireland

* **Correspondence**: To whom correspondence may be addressed. Email: [rwoods@ccrc.uga.edu](mailto:rwoods@ccrc.uga.edu), Voice: +1-706-542-4454, FAX: +1-706-542-4412

- 1. **Supplemental Methods**

## Details of BLI experiments

Measurements were set as 60s (equilibration) - 300s (activation) – 600s (immobilization) – 300s (quenching) – 120s (baseline) – 600s (association) – 600s (dissociation) at 25°C. For details about operation of BLI please see Octet BLI technical note 26 from ForteBio webpage. All BSA and BSA conjugates were prepared in analysis buffer at 1 µM concentration. scFv was loaded onto AR2G biosensor at 1 µM in water.

- 1. **Supplementary Tables**

## Supplementary Table 1. Results from preliminary docking experiments

| **Rank** | **Residue^a^** | **CHI Energy^b^** |
| --- | --- | --- |
| 1 | GalNAc | 6.9 |
| 2 | GalNAc | 4.4 |
| 3 | Gal | 4.5 |
| 4 | Fuc | 9.4 |
| 5 | Gal | 3.4 |
| 6 | Gal | 5.6 |
| 7 | Fuc | 9.1 |
| 8 | Gal | 9.9 |
| 9 | Fuc | 1.8 |
| 10 | Gal | 9.8 |
| 11 | GalNAc | 4.5 |
| 12 | Fuc | 2.2 |
| 13 | - | 3.3 |
| 14 | - | 9.0 |
| 15 | Gal | 2.1 |
| 16 | Gal | 6.0 |
| 17 | Gal | 4.0 |
| 18 | GalNAc | 3.9 |
| 19 | GalNAc | 5.0 |
| 20 | Gal | 9.4 |

^a^Indicates the residue located in the V-shaped binding pocket

^b^Energies of the conformation of the docked pose calculated using a CHI energy scoring function[^1^](#_ENREF_1). All values are in kcal/mol.

Supplementary Table 1

Twenty docked poses were generated using Autodock VINA. Each of the docked poses were scored using a CHI Energy scoring function. Docked poses with energy higher than 5kcal/mol were eliminated. Of the remaining docked structures, poses in which GalNAc was not within the V-shaped deep binding pocket were eliminated. The remaining docked poses (Rank 2,11,18 and 19) were subjected to MD simulations. None of these complexes remained stable.

## Supplementary Table 2. Binders from the glycan array screening of scFv AC1001 against the Consortium of Functional Glycomics (CFG) printed glycan array (v4.0). Complete data can be accessed on the CFG website (request ID: 1808)

| **Glycan Sequence** | **Experimental RFU^a^** |
| --- | --- |
| GalNAca1-3(Fuca1-2)Galb1-4GlcNAcb1-3Galb1-4GlcNAcb-Sp0 | 53921 |
| GalNAca1-3(Fuca1-2)Galb1-4GlcNAcb-Sp0 | 51949 |
| GalNAca1-3(Fuca1-2)Galb1-3GlcNAcb-Sp0 | 47339 |
| GalNAca1-3(Fuca1-2)Galb1-4GlcNAcb-Sp8 | 46920 |
| GalNAca1-3(Fuca1-2)Galb1-4Glcb-Sp0 | 41543 |
| GalNAca1-3(Fuca1-2)Galb-Sp8 | 37136 |
| GalNAca1-3(Fuca1-2)Galb1-4GlcNAcb1-3Galb1-4GlcNAcb1-3Galb1-4GlcNAcb-Sp0 | 36786 |
| GalNAca1-3(Fuca1-2)Galb1-4GlcNAcb1-2Mana1-3(GalNAca1-3(Fuca1-2)Galb1-4GlcNAcb1-2Mana1-6)Manb1-4GlcNAcb1-4GlcNAcb-Sp20 | 35917 |
| GalNAca1-3(Fuca1-2)Galb1-3GalNAca1-3(Fuca1-2)Galb1-4GlcNAcb-Sp0 | 35914 |
| GalNAca1-3(Fuca1-2)Galb1-4(Fuca1-3)GlcNAcb-Sp0 | 34747 |
| GalNAca1-3(Fuca1-2)Galb1-3GlcNAcb1-2Mana1-3(GalNAca1-3(Fuca1-2)Galb1-3GlcNAcb1-2Mana1-6)Manb1-4GlcNAcb1-4GlcNAcb-Sp20 | 34620 |
| GalNAca1-3(Fuca1-2)Galb-Sp18 | 33170 |
| GalNAca1-3(Fuca1-2)Galb1-4GlcNAcb1-3GalNAca-Sp14 | 30552 |
| GalNAca1-3(Fuca1-2)Galb1-3GlcNAcb1-3GalNAc-Sp14 | 29972 |
| Fuca1-2Galb1-3GalNAca1-3(Fuca1-2)Galb1-4GlcNAcb-Sp0 | 4748 |

^a^Relative Fluorescence Units from the array screening

## Supplementary Table 3. Comparison of theoretical (computational carbohydrate grafting) and experimental data for glycan array screening of scFv AC1001 against a carbohydrate array.

| **Glycan Sequence** | **CCG^a^** | **Experimental RFU^c^** | | | | |
| --- | --- | --- | --- | --- | --- | --- |
|  | **Score^b^** | **0.2^d^** | **2** | **20** | **100** | **200** |
| Cy3-BSA (20mg/mL + BSA, 125mg/mL total) | 0 | 79997 | 85412 | 111073 | 57627 | 93252 |
| GalNAca1-3[Fuca1-2]Galb1-4GlcNAcb-Sp-BSA | 0 | 4007 | 15442 | 16935 | 19376 | 31929 |
| GalNAca1-3(Fuca1-2)Galb1-3GalNAcb1-3Gala1-4Galb1-BSA | 0 | 4552 | 17206 | 22662 | 22679 | 22362 |
| GalNAca1-3(Fuca1-2)Galb1-3Galb1-linker-BSA | 0 | 3454 | 14997 | 18395 | 19402 | 22027 |
| GalNAca1-3(Fuca1-2)Galb1-4GlcNAcb1-linker-BSA | 0 | 4634 | 15633 | 17443 | 19354 | 21289 |
| GalNAca1-3[Fuca1-2]Galb1-3GlcNAcb-Sp-BSA | 0 | 4441 | 14639 | 17987 | 18327 | 20932 |
| GalNAca1-3(Fuca1-2)Galb1-3GlcNAcb1-linker-BSA | 0 | 5758 | 15841 | 19124 | 20324 | 20864 |
| GalNAca1-3(Fuca1-2)Galb1-4Glcb1-linker-BSA | 0 | 3927 | 15086 | 18092 | 19159 | 20785 |
| GalNAca1-3(Fuca1-2)Galb1-3GalNAcb1-linker-BSA | 0 | 4888 | 15188 | 17374 | 20457 | 20739 |
| GalNAca1-3(Fuca1-2)Galb1-3GalNAcα1-linker-BSA | 0 | 3302 | 12027 | 15352 | 19104 | 20220 |
| GalNAca1-3[Fuca1-2]Galb1-4[Fuca1-3]GlcNAcb-Sp-BSA | 0 | 5711 | 15196 | 18043 | 18027 | 18947 |
| GalNAca1-3(Fuca1-2)Galb- -BSA | 0 | 985 | 10291 | 15891 | 18071 | 17572 |
| use 25ug/mL + 100ug/mL BSA | 0 | 15926 | 11208 | 13943 | 8524 | 16557 |
| GalNAca1-3[Fuca1-2]Galb1-4GlcNAcb-Sp-BSA | 0 | 1306 | 9807 | 14607 | 13612 | 16062 |
| GalNAca1-3(Fuca1-2)Galb1-3GlcNAcb1-3Galb1-4(Glc)-APD-HSA | 0 | 1662 | 9483 | 12090 | 14604 | 15524 |
| GalNAca1-3[Fuca1-2]Galb1-3GlcNAcb-Sp-BSA | 0 | 2006 | 10983 | 20920 | 16997 | 15481 |
| GalNAca1-3(Fuca1-2)Galb1-3GalNAcb1-3Gala1-4Galb1-BSA | 0 | 722 | 7078 | 9496 | 15315 | 14869 |
| GalNAca1-3[Fuca1-2]Galb1-4[Fuca1-3]GlcNAcb-Sp-BSA | 0 | 4152 | 15498 | 23244 | 24923 | 14708 |
| GalNAca1-3[Fuca1-2]Galb1-4GlcNAcb-Sp-BSA | 0 | 978 | 8354 | 12292 | 13545 | 14688 |
| GalNAca1-3(Fuca1-2)Galb1-3(Fuca1-4)GlcNAcb1-3Galb1-BSA | 0 | 4957 | 11959 | 14146 | 15916 | 13625 |
| GalNAca1-3(Fuca1-2)Galb1-3GlcNAcb1-linker-BSA | 0 | 779 | 7088 | 11132 | 12175 | 12878 |
| GalNAca1-3(Fuca1-2)Galb1-3GalNAcb1-linker-BSA | 0 | 587 | 6762 | 11120 | 13016 | 12831 |
| GalNAca1-3(Fuca1-2)Galb1-4GlcNAcb1-linker-BSA | 0 | 343 | 4564 | 9932 | 11241 | 11679 |
| GalNAca1-3(Fuca1-2)Galb1-3Galb1-linker-BSA | 0 | 247 | 4306 | 8506 | 11066 | 11549 |

^a^Computational Carbohydrate Grafting[^2^](#_ENREF_2)

^b^Relative van der Waals overlap[^2^](#_ENREF_2)

^c^Relative Fluorescence Units from the array screening

^d^Concentration in μg/ml

- 1. **Supplemental Figures**

## Supplementary Figure 1


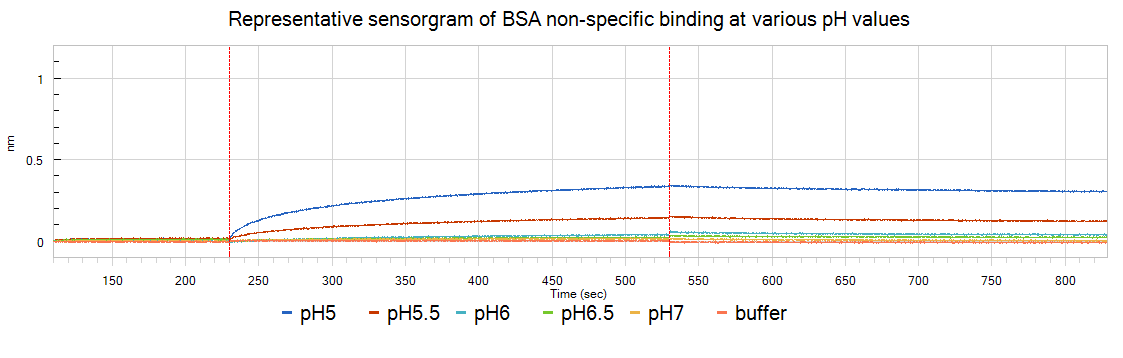


Supplementary Figure 1

Binding assay of BSA at various pH values. scFv-immobilized biosensor was dipped into 1 µM BSA at pH5 (blue), pH5.5 (red), pH6 (cyan), pH6.5 (green), pH7 (yellow), and buffer (orange). BSA showed no binding to scFv-immobilized biosensor at pH 6.5, and 7, but a relative small non-specific binding to scFv-immobilized biosensor at acidic pH 5, 5.5, and 6. Analysis buffer (reference in orange) did not display any binding at all.

## Supplementary Figure 2


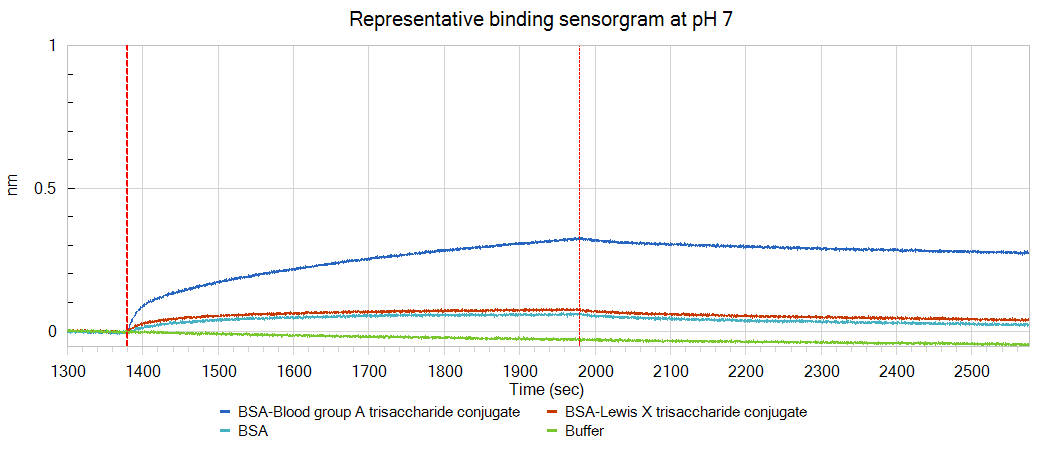


Supplementary Figure 2

Representative binding sensorgram for BGA-conjugate (blue), Le^x^-conjugate (red), BSA (cyan) and buffer (green) at pH 7. BSA-Le^x^ and BSA showed a similar signal.

## Supplementary Figure 3

##
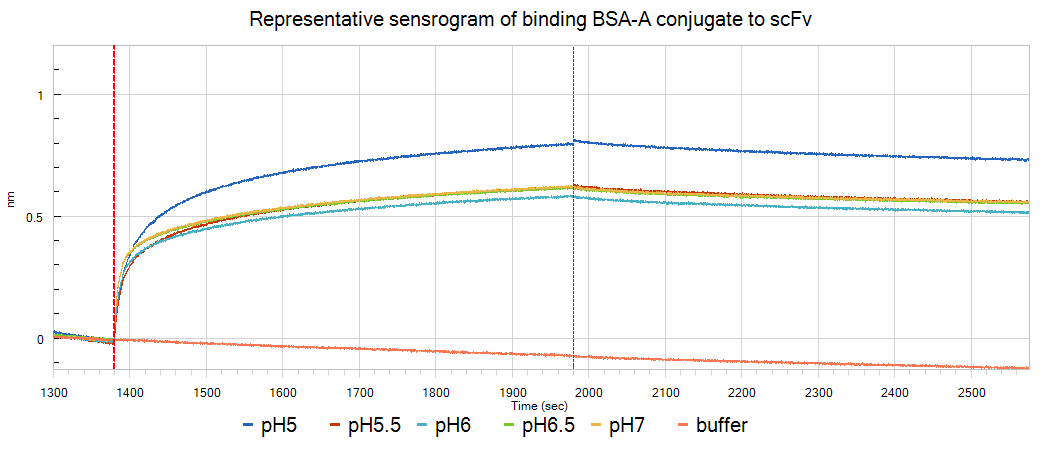


Supplementary Figure 3

BLI binding assay of scFv to BSA-blood group A trisaccharide conjugate at pH5 (blue), pH5.5 (red), pH6 (cyan), pH6.5 (green), pH7 (yellow), and buffer (orange).

1. **References**

1. Nivedha, A. K.; Makeneni, S.; Foley, B. L.; Tessier, M. B.; Woods, R. J., Importance of ligand conformational energies in carbohydrate docking: Sorting the wheat from the chaff. *Journal of Computational Chemistry* **2014,** *35* (7), 526-539.

2. Tessier, M. B.; Grant, O. C.; Heimburg-Molinaro, J.; Smith, D.; Jadey, S.; Gulick, A. M.; Glushka, J.; Deutscher, S. L.; Rittenhouse-Olson, K.; Woods, R. J., Computational Screening of the Human TF-Glycome Provides a Structural Definition for the Specificity of Anti-Tumor Antibody JAA-F11. *PLoS ONE* **2013,** *8* (1), e54874.
